# Characterization and optimization of antifungal production in *Streptomyces* sp. RMIT01 from the Australian mangrove rhizosphere

**DOI:** 10.7717/peerj.20901

**Published:** 2026-05-04

**Authors:** Sumali Lakmini Dissanayake Jayaweera, Daniel Anthony Dias, Chaitali Dekiwadia, Ben Wade, Thi Thu Hao Van

**Affiliations:** 1 Department of Biology, School of Science, Royal Melbourne Institute of Technology, Bundoora, Victoria, Australia; 2 ARC Training Centre for Hyphenated Analytical Separation Technologies (HyTECH), Deakin University, Burwood, Victoria, Australia; 3 CASS Food Research Centre, School of Exercise and Nutrition Sciences, Deakin University, Burwood, Victoria, Australia; 4 RMIT Microscopy & Microanalysis Facility, Royal Melbourne Institute of Technology, Melbourne, Victoria, Australia

**Keywords:** *Streptomyces* sp. RMIT01, Antifungal, *Candida albicans*, Mangrove rhizosphere, Biosynthetic gene clusters, Genome characterization, Species delineation

## Abstract

**Background:**

Mangroves are dynamic coastal ecosystems that provide a rich habitat for diverse microorganisms. *Streptomyces* strain RMIT01 was isolated from the rhizosphere of the mangrove *Avicennia marina* located in Jawbone Sanctuary, Williamstown, Victoria, Australia. This study aimed to characterize RMIT01’s genomic and biochemical traits, optimize its antifungal production, and explore its biosynthetic gene clusters to predict potential antifungal compounds.

**Methods:**

The RMIT01 strain was characterized using International *Streptomyces* Project (ISP) media series to observe morphological traits over a 21-day period, comparing results with standard species and colour charts. Micromorphological features were examined through light and scanning electron microscopy. Growth tolerance was assessed across various temperatures, pH levels, and NaCl concentrations in ISP2 medium, along with testing carbon and nitrogen source utilisation using standard kits. Whole genome sequencing was conducted using the Illumina platform, and AntiSMASH 8.0 was employed to predict secondary metabolite biosynthetic gene clusters, aiding in the identification of potential antimicrobial compounds.

**Results:**

The strain exhibited morphological and biochemical characteristics consistent with the genus *Streptomyces* possessing a Gram-positive cell wall and square shaped spores. Genomic analysis revealed a size of 7,609,141 bp and a Guanine and Cytosine (GC) content of 72%. The average nucleotide identity between strain RMIT01 and the closest related species- *Streptomyces sindenensis* JCM 4164^T^, *Streptomyces parvus* JCM 4069^T^, and *Streptomyces* YPW6 - were 92.19%, 92.45%, and 98.22%, respectively. Given the 95% cutoff for species delineation, RMIT01 is likely to represent a new species, as *Streptomyces* YPW6 has not yet been officially described or named. RMIT01 demonstrated antifungal properties against *Candida albicans*. Antifungal optimization experiments showed that incubation of RMIT01 in starch casein medium with Sigma TM sea salt at 25 °C for 6 days under aerobic conditions yielded a maximum inhibition zone diameter of 20.9 mm. The RMIT01 genome harbours unique biosynthetic gene clusters for bafilomycin B1; thiazostatin, watasemycins, and 2-hydroxyphenylthiazoline; enantiopyochelin and isopyochelin; tylactone; inthomycin; desulfoclethramycin/clethramycin; and niphimycin, several showing low similarity to known biosynthetic gene clusters.

**Conclusions:**

These findings highlight the significant potential of *Streptomyces* sp. RMIT01 as a source of antifungal agents, emphasizing the importance of exploring mangrove ecosystems for biotechnological applications.

## Introduction

The Gram-positive bacterial genus *Streptomyces* belongs to the phylum Actinomycetota and was introduced by Waksman & Henrici (1943). Currently, there are 787 validly published *Streptomyces* species excluding synonyms (Parte et al., 2020). *Streptomyces* are aerobic filamentous bacteria found in soil and, in some situations, associated with plants and animals (Huang et al., 2019; Jiang et al., 2020; Li et al., 2020). The genus *Streptomyces* is the predominant culturable genus within the phylum Actinomycetota. It is especially important due to its capability to produce a wide range of secondary metabolites, including antibacterial, antifungal, and antitumor compounds (Chen et al., 2020; Luo et al., 2024; Wang et al., 2024). The genus *Candida* is a yeast belonging to the family *Saccharomycetaceae* in the Ascomycota division of the Kingdom Fungi. *Candida* is part of the normal microbiota on skin and mucosal surfaces, including the gastrointestinal, respiratory and genitourinary tract (Köhler, Casadevall & Perfect, 2015). Under favourable conditions, these organisms can become opportunistic pathogens, causing diseases in individuals with impaired immunity, and can rapidly grow on the skin, nails, mouth, vagina and even within internal organs and the bloodstream. There are several species of *Candida* that can cause Candidiasis, with the most common being *Candida albicans*; other species include *Candida parapsilosis* and *Candida tropicalis* (Beardsley et al., 2018; Pfaller et al., 2010). Invasive *Candida* in the blood stream causes 1.57 million infections every year leading to a mortality rate of 63.6% (Denning, 2024). Reports from recent years indicate that fluconazole resistance of *C. albicans* has shown an increasing trend (Africa & dos Santos Abrantes, 2017; Beardsley et al., 2018; Nguyen & Ren, 2025).

The existing ability of a *Streptomyces* species to produce antifungal compounds can be enhanced or diminished by varying nutrient constituents and culturing conditions (Yang et al., 2025). Antimicrobial compound biosynthesis of *Streptomyces* is influenced by the source of carbon, nitrogen, and trace elements and culture conditions such as temperature, pH, and oxygen levels. Additionally, different species of *Streptomyces* respond differently to nutrient sources and fermentation conditions (Almeida et al., 2019; Lertcanawanichakul & Sahabuddeen, 2023; Yang et al., 2025). Under optimal conditions for secondary metabolite production, these strains have the potential to produce an enhanced number/quantity of bioactive compounds (Almeida et al., 2019; Ismet et al., 2004). Alongside optimization, predicting the genomic potential for secondary metabolites biosynthesis is important. Genome mining can reveal biosynthetic gene clusters (BGCs) encoding novel antifungal compounds, which is invaluable for prioritising strains with promising biosynthetic capacity, even when some metabolites are not expressed under standard laboratory conditions (Medema et al., 2015).

In this study, the *Streptomyces* strain RMIT01 was isolated from the mangrove *Avicennia marina* rhizosphere soil, collected from Jawbone Marine Sanctuary, Williamstown, Victoria, Australia. The sanctuary lies within an Aboriginal cultural landscape in the traditional Sea Country of the Bunurong Traditional Owners. This marine habitat comprises mangrove and saltmarsh plants within the Northern Port Phillip. Mangroves are terrestrial plants highly adapted to extremely saline, waterlogged and anaerobic soil conditions prevailing in coastal ecosystems and are rich sources of endophytic and rhizospheric microbes (Azman et al., 2015; Wainwright et al., 2023). The aims of this study were to characterize the strain RMIT01, including its genomic and biochemical characteristics and taxonomic assignment, optimize conditions for antifungal production, and perform genome mining to identify BGCs and predict their potential roles in antifungal compound production.

## Materials & Methods

### Isolation and maintenance of strain

All culturing was performed under aerobic conditions, unless otherwise specified.

The strain RMIT01 was isolated from the mangrove *A. marina* rhizosphere soil. The soil sample was air-dried at room temperature for 14 days, serial diluted, and spread on International *Streptomyces* Project (ISP) medium two, also called yeast extract-malt extract agar (Shirling & Gottlieb, 1966), with 1% malt extract, 0.4% glucose, 0.4% yeast extract, and 4% sea salt (Sigma™, St. Louis, MO, USA). The plates were incubated at 30 °C for three days. Purified colonies were identified using matrix-assisted laser desorption/ionization time-of-flight (MALDI-TOF) mass spectrometry (Bruker Daltonics, Bremen, Germany). Data were analysed using the Bruker MALDI Biotyper 4.1.80 software package; however, no identification was obtained (score value of 1.3). The isolates were stored in glycerol suspensions (35% v/v) at −80 °C for further experiments.

### Phenotypic characters

The strain was grown on ISP2 (Yeast malt agar), ISP3 (Oatmeal agar), ISP4 (inorganic salts-starch agar), and ISP5 (glycerol-asparagine agar) media with two replicates in order to observe morphological characters such as areal mycelium colour, substrate mycelium and, diffusible pigment colour (Shirling & Gottlieb, 1966). Observations were taken 7, 14, and 21 days after culture initiation from two replica plates following the method described in Shirling & Gottlieb (1966) comparing standard colour charts from the ISCC-NBS (Centore, 2016). Character comparisons were made using the species *Streptomyces badius* ISP 5139, *Streptomyces parvus* NRRL-B-1455, *Streptomyces sindenensis* ISP 5255, *Streptomyces bacillaris* INMI 445, and *Streptomyces griseus* IMRU 3463.

Mycelial and spore-forming characters were observed using micromorphological characterization methods, such as light and Scanning electron microscopy (SEM), at RMIT Microscopy and Microanalysis Facility, RMIT University. The RMIT01 strain was streaked on ISP3 media and clean glass coverslips were embedded in the culture at an angle of 45 degrees (Kurtböke, 2022) and incubated at 30 °C with three replicates. Sample preparation was done after 7, 14, and 21 days of culturing that represent each replicate. Coverslips were placed on a petri dish facing mycelial grown side upward and were conditioned using 100 µl of primary fixative in cacodylate buffer (2% paraformaldehyde and 2.5% glutaraldehyde in 0.1 M sodium cacodylate) for a few seconds. The fixative was rinsed thrice with 0.1 M sodium cacodylate buffer. A post-fixative solution (aqueous 1% OsO_4_) was then added for 15 min. The coverslip was rinsed twice with Milli Q water for three minutes each time. Samples were dehydrated with 50% acetone and 75% acetone solution for 15 min followed by addition of 100% acetone over the coverslips for complete dehydration. The acetone was evaporated inside the fume hood for one hour until the coverslips were completely dried. The samples were sputter coated with gold using a SPI Sputter Coater prior to imaging. Scanning electron microscopic images were acquired using an FEI Quanta 200 SEM.

Temperature tolerance for growth of RMIT01 at 4, 10, 15, 20, 25, 30, 35, 37, 40, and 45° C was evaluated for 14 days (Li et al., 2019). Tolerance for the pH was determined with different pH levels from 4–11 and incubated at 28° C for 14–21 days. Buffer systems were used for different pH range as described previously (Xie et al., 2012). The NaCl (0–10%) were determined for 14–21 days at 28 °C (Komaki et al., 2020). All three tests were done on ISP2 medium.

Carbon source utilisation was examined using the API^®^ 50 CHB/E test kit (Biomerieux™, Marcy-l’Étoile, France) by following the product instructions. Compounds that can be utilised as a sole nitrogen source were tested using the method described in Williams et al. (1983). The tested nitrogen sources included were L-cysteine, L-lysine, L-glycine, L-glutamine, L-valine, L-arginine, L-asparagine, and skim milk. Other standard biochemical characterization tests such as the liquefaction of gelatin, hydrolysis of starch, hydrolysis of Tween (Tween 20 and 80), reduction of nitrate, production of H_2_S, and catalase production were performed as described by Frazier (1926) and Williams et al. (1983). Antibiotic susceptibility was determined using the freeze-dried filter paper disc method as described by Williams et al. (1983).

### Genomic characteristics

DNA extraction was performed using the method described by Das et al. (2024) which was a modified method of Mayjonade et al. (2016). The whole genome of RMIT01 was sequenced using Illumina MiSeq platform. The genome assembly was performed using the A5-miseq pipeline on a 64-bit Linux computer operating system (Coil, Jospin & Darling, 2014). The resultant FASTA sequence was used for genome analysis *via* Rapid Annotation using Subsystem (RAST) Version 2.0 (Overbeek et al., 2014). The output was used to obtain the 16s rRNA sequence and Basic Local Alignment Search Tool (BLAST) available at https://blast.ncbi.nlm.nih.gov/Blast.cgi (Altschul et al., 1990) was used to search for similarity between 16s rRNA sequences available on the NCBI database. The Type (Strain) Genome Server (TYGS) available on https://tygs.dsmz.de (Meier-Kolthoff & Göker, 2019) was also used to identify closely related type strains. Phylogenetic relationships were inferred with FastME 2.1.6.1 (Lefort, Desper & Gascuel, 2015), based on Genome BLAST Distance Phylogeny (GBDP) distances calculated from whole genome sequences. Whole genome sequences of related species’ type strains were obtained from the NCBI database (https://www.ncbi.nlm.nih.gov/datasets/genomes/) and were annotated using RAST. The RAST annotated outputs and NCBI GenBank records were used to obtain housekeeping gene sequences, namely, *atpD* (ATP synthase beta subunit), *gyrB* (DNA gyrase B subunit), *recA* (recombinase A), *rpoB* (RNA polymerase beta subunit), and *trpB* (tryptophan synthetase, beta subunit) of RMIT01 and type strains of related species. Equal sized fragments (1,354 bp) of 16S rRNA sequences were trimmed manually. The five housekeeping gene sequences *atpD*, *gyrB*, *recA*, *rpoB*, and *trpB* of RMIT01 were also trimmed creating segments of 496, 408, 491, 540, and 571 bp, respectively. These were concatenated by joining them head to tail in the above order, creating a 2,506 bp multi-locus sequence. The same method was repeated for the type strains. Further, the prepared gene sequences were aligned separately using Multiple Sequence Comparison by Log-Expectation (MUSCLE v5) (Edgar, 2004) before the following analyses. Housekeeping gene sequences were used to estimate pairwise genetic distance (Multi Locus Sequence Analysis - MLSA) using the Kimura 2-parameter model (Kimura, 1980) available in Molecular Evolutionary Genetics Analysis (MEGA) 12 software (Kumar et al., 2024). Phylogenetic trees based on 16S rRNA and housekeeping gene sequences were constructed using neighbour-joining (Saitou & Nei, 1987) tree-making algorithms with 1,000 bootstrap tests (1,000 replicates) (Felsenstein, 1985) using the same software.

The Average Nucleotide Identity (ANI) values were calculated using MUMmer-based approaches (ANIb and ANIm in a reciprocal best hits) and the OrthoANIu algorithm *via* https://jspecies.ribohost.com/jspeciesws/#home and https://www.ezbiocloud.net/tools/ani, respectively (Richter et al., 2016; Yoon et al., 2017).

### Antimicrobial susceptibility testing and antifungal production optimization

#### Inoculation of RMIT01 culture on the basal media

Antifungal susceptibility testing was conducted using a gel diffusion bioassay with *C. albicans* strain American Type Culture Collection (ATCC) 10251 as the indicator strain. The test culture was prepared in three replicates of 7 mL starch casein (SC; 0.1% soluble starch and 0.003% casein sodium salt with 4% Sigma™ sea salt, pH 7.8) broth media in 15 mL falcon tubes. Each replicate was inoculated with a single 2 mm diameter colony of RMIT01 from a SC plate culture and incubated at 30 °C for 3 days. Subsequently, 20 µL of broth culture was used as the inoculum to apply on SC media plate. The OD_600_ value of the 5-folds diluted sample was between 0.7 and 0.8. The inoculum was uniformly applied on each SC media plate using a sterile spreader and incubated at 30 °C.

#### *C. albicans* inoculum preparation

A starter culture of *C. albicans* was prepared 24 h prior to the bioassay. The organism was maintained at 37 °C on Sabouraud dextrose agar (SDA - dextrose 4%, peptone 1% and pH 5.6). A single colony was streaked onto a fresh SDA plate and incubated under the same conditions. One mm diameter *C. albicans* colonies were suspended in 8 mL of sterile 0.145-mol/L saline, comparable to 1.5 times of the 0.5 Mcfarland Standard. A square shape bioassay plate (bioassay dishes CLS431111-16EA - 245 mm × 245 mm × 18 mm; Corning^®^ Square, Corning, NY, USA) was prepared by using 200 mL of Roswell Park Memorial Institute Medium RPMI 1640 with 0.165 M 3-(N-Morpholino) propanesulfonic acid (Wayne, 2002) and 2% glucose consisting gel media (pH 7). The indicator strain was lawn cultured using the prepared *C. albicans* inoculum.

#### *Streptomyces* gel disc preparation

A *Streptomyces* plate from each replicate and a blank SC agar plate, was used for the antifungal bioassay test. Gel discs were prepared using a flame-sterilized cork borer (9 mm diameter). From this step onwards, two technical replicates of gel discs were prepared from each biological replicate, with a total of six replicates. The *Streptomyces* colony growing on each gel disc was carefully sliced off and discarded.

#### Antifungal assay and optimization

A negative control was prepared by excising a gel disc from an uninoculated SC media. For the positive control, 2 µL of 1 µg/µL fluconazole in water was added into a SC gel disc and allowed to fully absorb. The *Streptomyces* gel discs were placed on the bioassay plate using sterile forceps with the gel disc facing upward. Bioassay plates were then incubated at 37 °C for 24 h. After incubation, the diameter of the inhibition zone was measured along two perpendicular directions and averaged.

To determine the effect of incubation time on antifungal activity, 15 SC plates were inoculated for each replicate, alongside uninoculated negative controls. All plates were incubated at 30 °C for 1 to 15 days. Every day, one plate per replicate was subjected to the antifungal bioassay following the procedure described above. One-variable-at-a-time was used to optimize each parameter and fixed before testing the next parameter as described below in the order they were undertaken.

The influence of the incubation temperature on the growth and antifungal production of RMIT01 was evaluated at 15 °C, 20 °C, 25 °C, 30 °C, 37 °C, and 40 °C with revised condition of 6 days of incubation time. Based on the result, 25 °C was used as the incubation temperature for all subsequent assays.

Media types were selected based on their ability to produce antifungals in literature (Augustine et al., 2004; Ismet et al., 2004; Mincer, Fenical & Jensen, 2005): M1 (1% soluble starch, 0.4% yeast extract, and 0.2% peptone) (Mincer, Fenical & Jensen, 2005), ISP2, SC and the high-antifungal produced media (1% glucose, 1% glycerol, 1% soluble starch, 0.25% corn steep powder, 0.5% peptone, 0.2% yeast extract) described by Ismet et al. (2004). For all the media 4% Sigma™ sea salt was added. SC media was used in all subsequent assays. The effect of various salt formulations such as 4% Tropic Marine™ salt, 4% Sigma™ sea salt, and an artificial sea salt mixture (0.48 M NaCl, 0.01 M KCl, 0.027 M MgCl_2_, 0.03 M MgSO_4_, 0.01 M CaCl_2_ and 0.002 M NaHCO_3_) described by Wilt & Benson (2004) were also examined with the selected media which was SC. 4% Sigma™ sea salt was used in all subsequent assays.

Additionally, the impact of different initial pH values of the media on which RMIT01 was grown (4.5, 5, 6, 7, 7.8, 9) were tested. Aerobic *versus* anaerobic conditions were investigated with an altered pH of 6. The anaerobic condition was generated in an anaerobic jar with a AnaeroGen™ gas pack, while aerobic cultures were incubated in the same incubator without an anaerobic jar. The inhibition zone diameter from each experimental treatment was analysed using GraphPad Prism software version 8 (GraphPad Software, Boston, MA, USA). A one-way analysis of variance (ANOVA) was performed for all the optimizing factors, except for the respiration method, which used an unpaired, two-tailed *t*-test. Tukey’s multiple comparisons test was applied following ANOVA when a significant effect was detected (*p* < 0.05) to identify treatments that differed significantly.

### Prediction of secondary metabolism using genome mining tools

AntiSMASH 8.0 (Blin et al., 2025) was used with standard parameters to analyse secondary metabolite BGCs of RMIT01, *Streptomyces* sp. YPW6, *S. parvus* JCM 4069^T^, and *S. badius JCM* 4350^T^.

## Results

### Characterization studies

Based on morphological characterization studies, the aerial spore mass colour of *Streptomyces* isolates was light yellow to greyish green yellow and the substrate mycelium was yellowish white to greyish greenish yellow on oatmeal agar, inorganic salts-starch agar and glycerol-asparagine agar (Table S1). No pigments were produced with any of the media.

**Figure 1:**
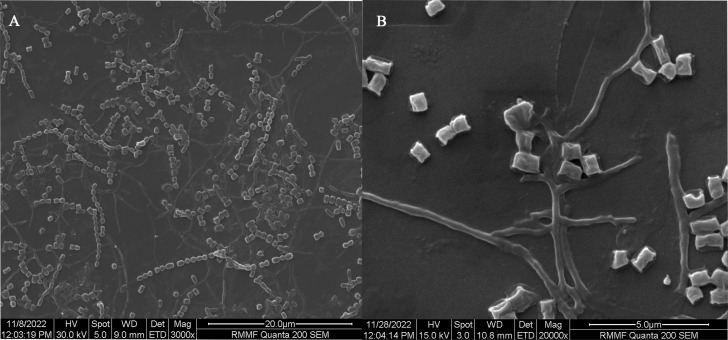
SEM micrographs of *Streptomyces* RMIT01. (A) RF spore chain at x3000 from seven-day old culture on oatmeal agar. (B) Culture mycelium with smooth square shaped spores at x20000 from 14-day old culture on oatmeal agar.

Microscopic examination of RMIT01 (Fig. 1 and Table S1), using SEM, exhibited branched mycelium and rectiflexibiles (RF) spore chains containing 15–20 smooth, square-shape spores formed from fragmentation of aerial mycelium grown on oatmeal agar after 1–2–weeks of incubation at 30 °C. D-xylose, D-mannose, D-mannitol and D-arabitol were utilised as carbon sources, while growth on esculin ferric citrate was also positive. Further, L-cysteine, L-lysine, L-glycine, L-glutamine, L-valine, L-arginine, and L-asparagine were utilised as nitrogen sources (Table S1). Additionally, 3% H_2_O_2_, Tween 20 and Tween 80 tests confirmed that RMIT01 produce catalase and lipase/esterase enzymes, respectively. Furthermore, RMIT01 is susceptible to tetracycline, kanamycin, streptomycin, lincomycin and vancomycin at all concentrations tested (1,000, 500, 100, 50, 10, 1 µg/mL).

### Genome analysis

Whole genome sequencing followed by genome assembly generated a genome size of 7,609,141 bp for RMIT01, with a Guanine and Cytosine (GC) content of 72% (Table 1). The genome sequence of the *Streptomyces* sp. RMIT01 strain has been deposited in the NCBI GenBank database under accession number CP194014. The ANI value between RMIT01 and *Streptomyces* sp YPW6 were the highest (ANIb, ANIm, and OrthoANIu were 97.88%, 98.26%, and 98.22% respectively), exceeding the species-level ANI threshold of 95–96% (Yoon et al., 2017) (Table 1).

**Figure 2:**
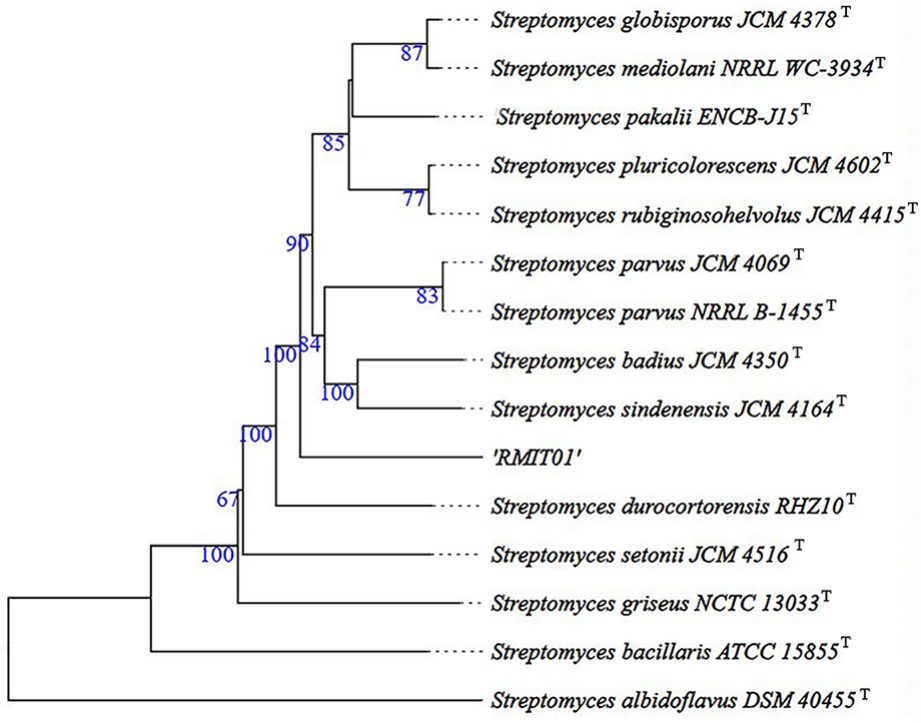
Whole-genome sequence-based dendrogram of closely related species generated using TYGS analysis. The branch lengths were scaled in terms of GBDP distance formula d_5_ (Meier-Kolthoff et al., 2013). The numbers above branches were GBDP pseudo-bootstrap support values >60% from 100 replications, with an average branch support of 85%. The tree was rooted at the midpoint (Farris, 1972).

The 16S rRNA gene sequence of RMIT01 based BLAST analysis revealed the following species: *Streptomyces* sp. JL1001 (CP136798.1), *Streptomyces* sp. SCSIO-PteL053 (CP059136.1), *Streptomyces sp*. S063 (CP021707.1), *Streptomyces* sp. Tue6075 (CP010833.1) and *Streptomyces* sp. SH5 (CP123780.1) displayed 99.78% nucleotide identity. Additionally, *Streptomyces* sp. JUS-F4 (CP098783.1), *Streptomyces* sp. SM1P (CP149585.1), *Streptomyces* sp. 1268 (CP162609.1), *S. parvus* strain F-G-2 (CP135079.1), *Streptomyces* sp. WA6-1-16 (CP084357.1), *Streptomyces* sp. YPW6 (CP076457.1), *S. parvus* strain A612 (CP159802.1) and, *Streptomyces* sp. CB04723 (CP058556.1) all shared 99.70% nucleotide identity with RMIT01. A whole-genome sequence-based dendrogram generated using TYGS analysis with closely related type strains is displayed in Fig. 2. The dendrogram shows that RMIT01 was not clustered together with any other closely related type strains of *Streptomyces*. According to the dendrogram, the most closely related type strains to RMIT01 were *S. sidenensis, S. badius, Streptomyces durocortorensis* and *S. parvus*.

**Table 1:** Genome size, GC content, and ANI values of strain RMIT01 and related *Streptomyces* species/strains.

**Species/Strain**	**NCBI/Genbank accession**	**Whole genome size (bp)**	**GC content**	**ANIb (%)** (Richter et al., 2016)	**ANIm (%)** (Richter et al., 2016)	**Ortho ANIu (%)** (Yoon et al., 2017)	**Interpretation**
RMIT01	CP194014	7,609,141	72%	N/A	N/A	N/A	N/A
*Streptomyces* sp. YPW6	CP076457.1	7,695,589	72.0%	97.88	98.26	98.22	Same species (The species name has not been formally assigned)
*S. parvus* JCM 4069^T^	NZ_BMRY00000000.1	8,032,512	71.5%	92.15	93.16	92.45	Different species
*S. badius* JCM 4350^T^	BMSZ00000000.1	7,515,187	71.7%	91.81	92.87	92.06	Different species
*S. bacillaris* ATCC 15855^T^	CP029378.1	7,888,441	72%	86.69	89.11	86.80	Different species
*S. durocortorensis* RHZ10	JAFEUF010000614.1	7,959,868	71.7%	90.77	92.29	91.32	Different species
*S. griseus* JCM 4516^T^ (type strain of *Streptomyces setonii*)	NZ_BNBJ01000001.1	8,065,044	71.9%	90.08	91.34	90.11	Different species
*S. sindenensis* JCM 4164^T^	BMSG00000000.1	7,564,582	71.8%	92.05	92.92	92.19	Different species
*Streptomyces* sp SM1P	CP149585.1	7,561,428	71.7%	92.58	93.46	92.96	Different species
*S. parvus* strain F-G-2	CP135079.1	7,143,830	71.19%	92.25	93.33	92.73	Different species

**Notes.**

N/A, Not applicable.

**Figure 3:**
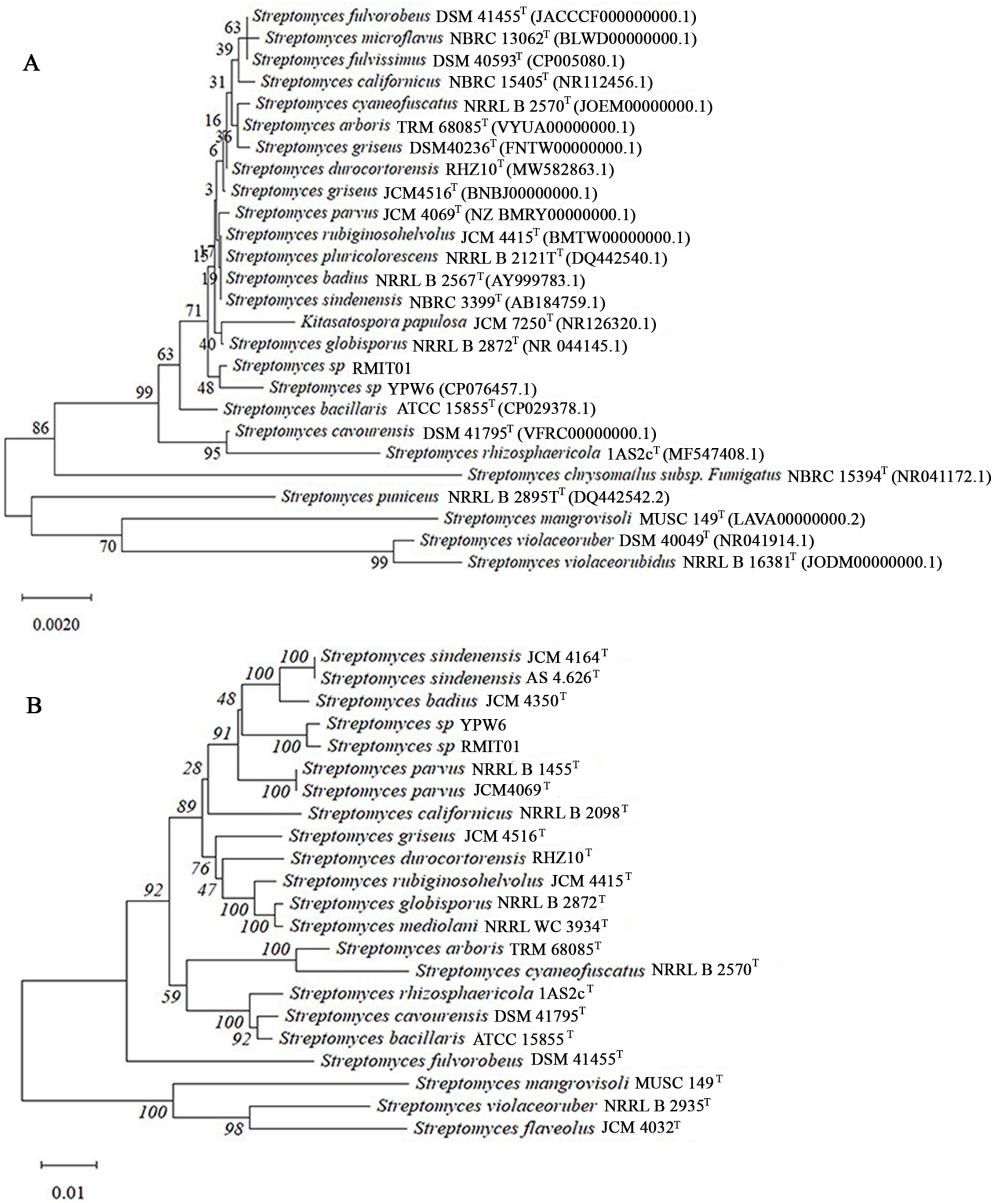
Neighbour joining phylogenetic trees of RMIT01 and related species using MEGA 12 (Kumar et al., 2024; Saitou & Nei, 1987). (A) 16S rRNA gene sequences (1,354 bp) based optimal tree with the sum of branch length = 0.088 is shown. Bootstrap values based on 1,000 replications are listed as percentage (Felsenstein, 1985). Bar: 0.002 nucleotide substitutions per site and pairwise deletion option was applied. (B) Five housekeeping gene sequences (2,506 bp nucleotides) based optimal tree with the sum of branch length = 0.390 is shown. Bootstrap values based on 1,000 replications are listed as percentages next to the branches (Felsenstein, 1985). Bar: 0.01 nucleotide substitutions per site and pairwise deletion option was applied.

16S rRNA sequence-based and five housekeeping gene-based neighbour-joining phylogenetic trees generated using MEGA 12 software are displayed on Figs. 3A and 3B respectively. The 16S rRNA sequence-based neighbour-joining phylogenetic tree (Fig. 3A), showed that strain RMIT01 formed a subcluster with *Streptomyces* sp YPW6 isolate, supported by 48% bootstrap values. According to the five housekeeping gene-based dendrogram (Fig. 3B), RMIT01 and *Streptomyces* YPW6 clustered in the same clade, supported by 100% bootstrap values and *S. sindenensis*, *S. badius*, and *S. parvus* were clustered on adjacent branches. MLSA analysis using five housekeeping genes (*atpD*, *gyrB*, *rpoB*, *recA* and *trpB*) (Table S2) showed that the genetic distance between RMIT01 and *Streptomyces* sp YPW6 was 0.00521, which is below the species delimitation threshold of 0.007 (Rong & Huang, 2014). In contrast, all other closely related species displayed MLSA genetic distance greater than 0.007 when compared with RMIT01.

### RMIT01 antifungal production testing and optimization

**Figure 4:**
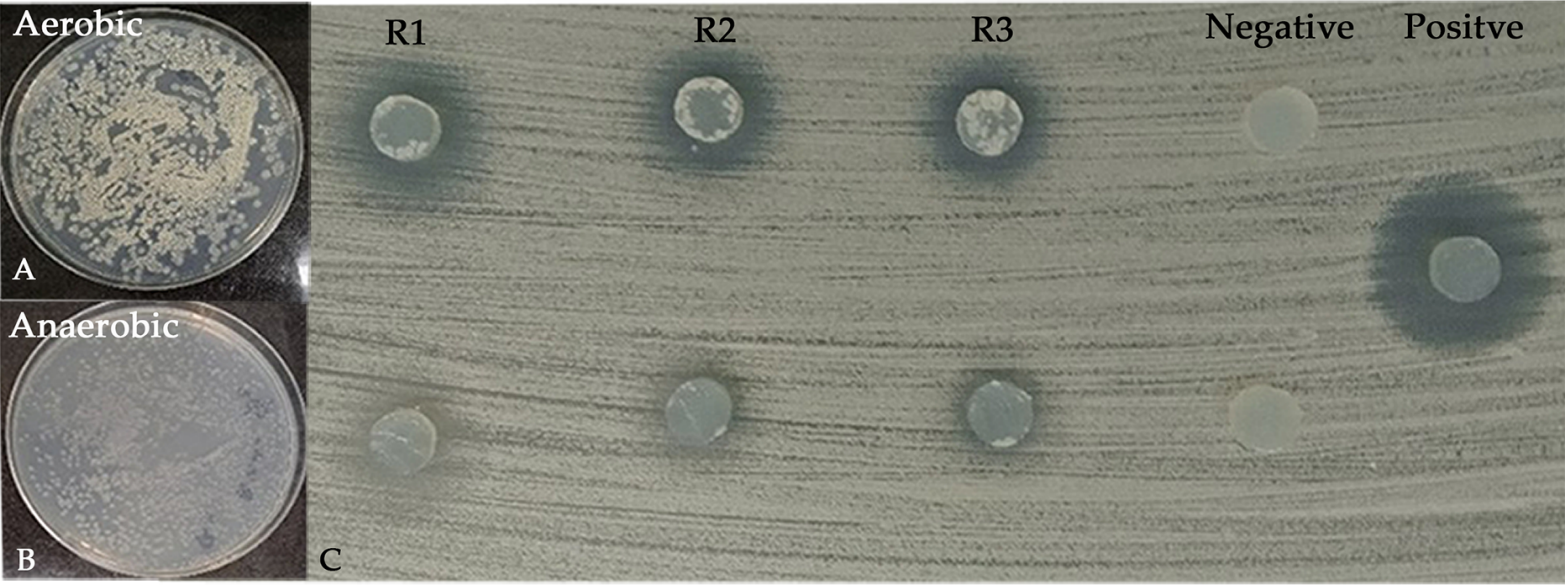
Agar plates that displays the growth of RMIT01 under aerobic and anerobic conditions and the corresponding antifungal activity against *C. albicans*, demonstrating the effect of aerobic and anaerobic conditions on antifungal production. (A) Growth of RMIT01 on SC agar plate aerobic conditions. (B) Growth of RMIT01 on SC agar under anaerobic conditions. (C) *C. albicans* bioassay plate showing agar gel discs prepared from aerobically grown cultures (top row) and anaerobically grown cultures (bottom row).

**Figure 5:**
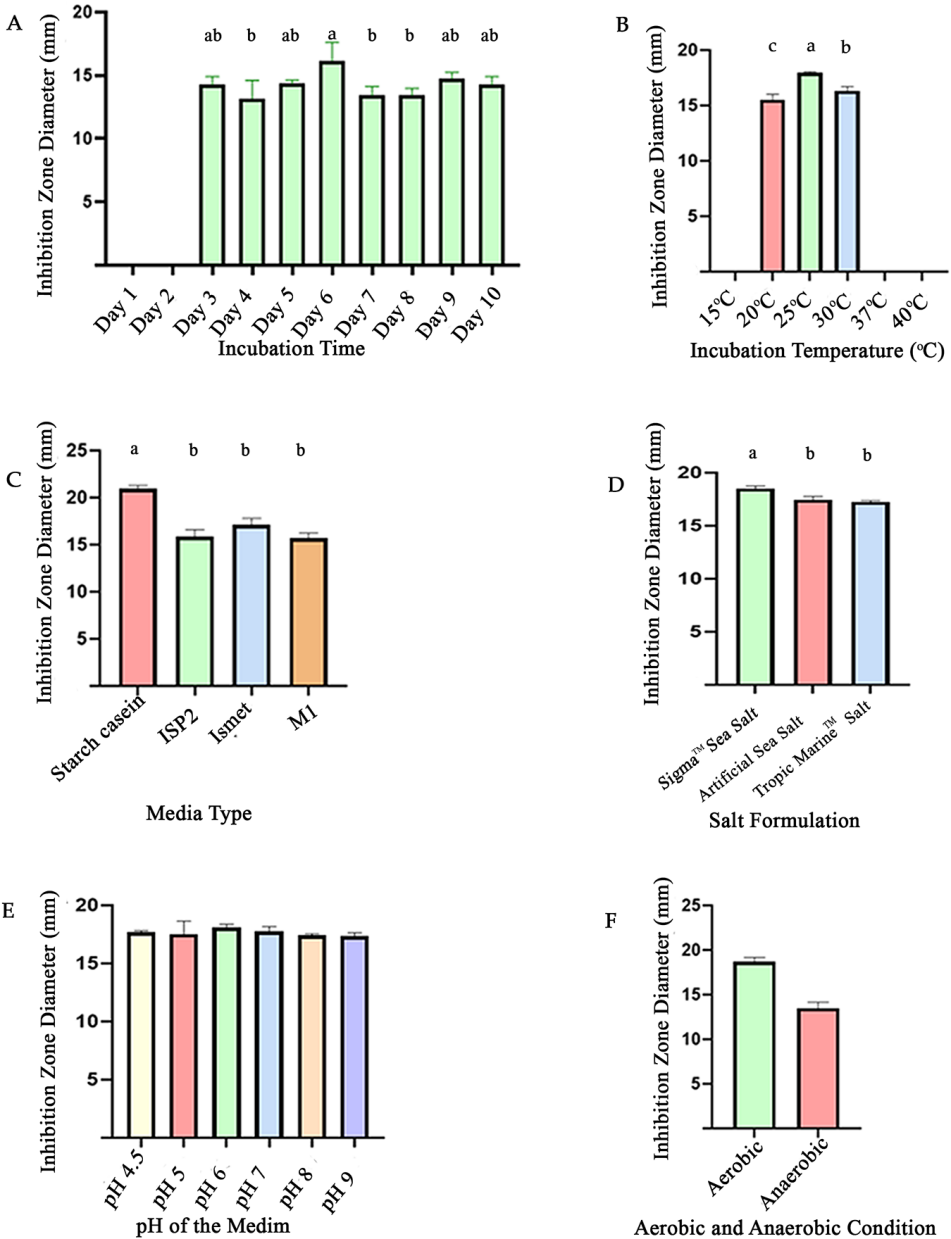
Effects of different culture conditions on antifungal production of RMIT01. (A) Incubation period, (B) incubation temperature, (C) media type, (D) salt formulation, (E) initial medium pH, (F) aerobic and anaerobic condition. Error bars represent standard deviation. (A, B, C, and D) analysed using one-way ANOVA followed by Tukey’s multiple comparisons, different letters above bars indicate significant differences (*p* < 0.05), while bars sharing at least one letter are not significantly different. (E) (pH) showed no significant differences by one-way ANOVA (*p* > 0.05). For (F), significance was assessed using an unpaired *t*-test (*p* < 0.05).

RMIT01 displayed antifungal activity against *C. albicans*. One-way ANOVA revealed significant treatment effects for at least one treatment from each experiment, such as incubation time (*p* = 0.0166), incubation temperature (*p* < 0.0001), media type (*p* < 0.0001) and salt composition (*p* = 0.0016) whereas the initial medium pH (*p* = 0.5851) had no significant effect on inhibition zone diameter. Figure 4 demonstrates that growth under aerobic conditions was stronger than under anaerobic conditions. The unpaired *t*-test for the aerobic *versus* anaerobic conditions experiment indicated a significant effect of respiration mode on antifungal activity (*p* = 0.0002), with aerobic cultures producing larger inhibition zone (mean = 18.7 mm) than anaerobic cultures (Figs. 4 and 5).

In the incubation time experiment, 6 days of incubation yielded the highest inhibition zone diameter (mean: 16.2 mm), which was significantly greater than those observed at 4 days (*p* = 0.0130), 7 days (*p* = 0.0255) and 8 days (*p* = 0.0255) (Fig. 5 and Table S3). In the temperature optimization experiment, incubation at 25 °C was recorded as producing the largest inhibition zone, significantly different (*p* < 0.0001 for both mean comparisons) from the adjacent temperatures of 20 °C and 30 °C (Fig. 5). Cultures of RMIT01 grown at 37 °C showed strong growth on plate, but no antifungal inhibition zone was observed. Further, incubation at 15 °C and 40 °C yielded very few bacterial colonies, even though the gel discs were cut covering the available colonies, no inhibition zones were reported.

Among the tested media types, SC agar produced the largest inhibition zone (mean = 20.9 mm), which was significantly greater than those observed for ISP2, M1 (*p* < 0.0001) and Ismet (*p* = 0.0003) media. For salt composition, the medium containing Sigma™ salt mixture yielded the largest inhibition zone (mean = 18.5 mm), which was significantly higher from those with artificial sea salt (*p* = 0.0046) and Tropic Marine salts (*p* = 0.0018). Both aerobic and anaerobic conditions supported bacterial growth (Fig. 4). However, cultures grown under anaerobic conditions exhibited a lighter appearance compared with those grown aerobically and produced a smaller inhibition zone diameter.

### AntiSMASH programme predicted BGCs

The AntiSMASH programme allowed us to identify and compare the potential secondary metabolites produced by these strains, providing insights into their biosynthetic capabilities and the possibility of novelty of compounds that may be associated with RMIT01. The analysis identified 47 BGCs within the genome of RMIT01 (169 scaffolds). Of these, 23 BGCs were matched to previously characterized BGCs in the MIBiG database (Medema et al., 2015), indicating they are well-known and better studied. Table 2 outlines these 23 gene clusters showing high (80–100%), medium (50–79%), and low (<50%) similarity to known BGCs. The other 24 unknown BGCs such as lanthipeptide, azole-containing-RiPP, polyketide synthase, and terpene showed no significant similarity to any reference BGCs and may represent putative or novel BGCs with the potential to encode unexplored secondary metabolites. Among the known clusters, BGCs responsible for the synthesis of desferrioxamin B, ectoine, geosmin, and AmfS exhibited high similarity to genome regions of RMIT01, and the closely related species evaluated. Clusters synthesising streptamidine and hopene displayed medium-similarity, whereas aborycin, coelimycin and 14-hydroxyisochainin showed low similarity to BGCs detected in RMIT01 as well as in its related species. Furthermore, predicted biosynthetic gene clusters responsible for synthesising bafilomycin B1; thiazostatin, watasemycin A, watasemycin B, and 2-hydroxyphenylthiazoline; enantiopyochelin and isopyochelin; tylactone; inthomycin (low similarity to known BGCs); desulfoclethramycin and clethramycin (low similarity to known BGCs); and niphimycin (low similarity to known BGCs) were unique to the RMIT01 genome compared with closely related *Streptomyces* species.

**Table 2:** Predicted BGCs in RMIT01 and related species matched to the MIBiG database using AntiSMASH.

**Gene cluster/Cluster type/Known BGC align with**	**RMIT01**	***Streptomyces*** ** YPW6**	***S. parvus*** ** JCM 4069** ^**T**^	***S. sindenensis*** ** JCM 4164** ^**T**^	***S. badius*** ** JCM 4350** ^**T**^
Desferrioxamin B/NI-siderophore/*Streptomyces griseus* subsp. griseus NBRC 13350	High	High	High	High	High
Ectoine/Ectoine/*Streptomyces anulatus*	High	High	High	High	High
Geosmin/Terpene/*Streptomyces coelicolor* A3	High	High	High	High	High
Naringenin (flavonoid)/T3PKS/ *Streptomyces clavuligerus* ATCC 27064	High		High	High	High
Coelichelin/NRP-metallophores, NRPS/*Streptomyces coelicolor* A3	High	High		High	High
2-methoxy-5-methyl-6-(13-methyltetradecyl)-1,4-benzoquinone/2-methoxy-5-methyl-6-(13-methyltetradecyl)phenol/T3PKS/*Streptomyces griseus* subsp. griseus NBRC 13350	High	High	High		
Melanin/Terpene-precursor, melanin/*Streptomyces griseus* subsp. griseus NBRC 13350	High	High		High	High
Bafilomycin B1/T1PKS/textitStreptomyces lohii	High				
AmfS/Lanthipeptide-class-iii/*/Streptomyces griseus* subsp. griseus NBRC 13350	High	High	High	High	High
Keywimysin/Lassopeptide/*Streptomyces* sp. NRRL F-5702	High			High	
10-epi-HSAF, 10-epi-3-deOH-HSAF,10-epi-maltophilin,10-epi-xanthobaccin C, 10-epi-hydroxymaltophilin, 10-epi-FI-2/T1PKS, NRPS/*Streptomyces* sp.	High	High			High
Thiazostatin, watasemycin A, watasemycin B, 2-hydroxyphenylthiazoline enantiopyochelin, isopyochelin/NRPS/*Streptomyces venezuelae* ATCC 10712	Medium				
Hopene/Terpene/*Streptomyces coelicolor* A3	Medium	Medium	Medium	Medium	Medium
Streptamidine/RiPP-like*/Streptomyces albidoflavus* J1074	Medium	Medium	Medium	Medium	Medium
Isorenieratene/Terpene*/Streptomyces griseus* subsp. griseus NBRC 13350	Medium	High	High		High
Tylactone/T1PKS/*Streptomyces fradiae*	Medium				
Aborycin/Hydrogen-cyanide/*Streptomyces* sp. ZS0098	Low	Low	Low	Low	Low
Showdomycin/Ectoine, butyrolactone*/Streptomyces showdoensis*	Low	Low		Low	Low
Inthomycin B/NRPS*/Streptomyces* sp.	Low				
Coelimycin P1/Butyrolactone/*Streptomyces coelicolor* A3	Low	Low	Low	Low	Low
Desulfoclethramycin/clethramycin/T1PKS, terpene/*Streptomyces* sp.	Low				
14-hydroxyisochainin/RiPP like*/Streptomyces peucetius* subsp. caesius ATCC 27952	Low	Low	Low	Low	Low
Niphimycin/T1PKS/*Streptomyces* sp. IMB7-145	Low				

**Notes.**

High similarity → usually considered ≥ 80% to a known BGC (very likely the same metabolite).

Medium similarity → roughly 50–80% (shared core genes but with differences, possible analogues).

Low similarity → <50% (only partial overlap; often indicates novelty).

T3PKS Type III Polyketide Synthase T1PKS Type I Polyketide Synthase NRPS Non-ribosomal Peptide Synthetase HSAF Heat-Stable Antifungal Factor

## Discussion

In this study, *Streptomyces* sp. RMIT01 was isolated from the rhizosphere of *A. marina*, a mangrove plant along the coastline of Jawbone Marine Sanctuary, Australia. The investigation focused on characterization of the *Streptomyces* strain, antifungal properties and genome mining. Morphological, physiological, and genomic characteristics were examined for taxonomic identification. DNA sequence analysis was used to determine the genetically closest related species.

In comparison to the genome size of related species, *S. sindenensis* JCM 4164^T^ exhibited the closest genome size (7,564,582 bp) to the RMIT01 and *Streptomyces* sp SM1P and *Streptomyces* sp. YPW6 showed the next closest genome sizes 7,561,428 and 7,695,589 bp respectively (Table 1). The GC content of *S. bacillaris* ATCC 15855^T^ and *Streptomyces* sp. YPW6 were similar to that of RMIT01 (72%, Table 1). Such similarities in genome size and GC content may reflect close phylogenetic relationships among these species.

The 16SrRNA phylogenetic tree showed that *Streptomyces* YPW6 is most closely related to RMIT01, with a bootstrap value of 48%. The low bootstrap support is likely due to the limited discriminatory power of the highly conserved 16S rRNA gene at the species level and the distance-based nature of the neighbour-joining method, which may be insufficient to resolve relationships among closely related *Streptomyces* taxa. However, the phylogenetic tree based on five housekeeping genes also showed that these two strains are most closely related, with high bootstrap support (100%). Dendrogram analysis indicated that *Streptomyces* YPW6, *S. sindenensis*, *S. badius*, and *S. parvus* are closely related species to *Streptomyces* sp. RMIT01. This relatedness was further supported by ANI analysis using the OrthoANIu algorithm, ANIb and ANIm, with values ≥95% indicating the same species and values <95% indicating different species (Richter & Rosselló-Móra, 2009). The ANI value between RMIT01 and *S. parvus* JCM 4069^T^, *S. sindenensis* JCM 4164^T^, and *S. badius* strain JCM 4350^T^ were 92.45%, 92.19%, and 92.06%, respectively, indicating that RMIT01 does not belong to any of the closest known species (Table 1). The ANI value between RMIT01 and *Streptomyces* YPW6 (CP076457.1) was 98.22%, exceeding the species delineation threshold. This was an NCBI entry submitted by the Marine Science and Technology College, Zhejiang Ocean University, Zhejiang, China, but has not been formally named (Qu, 2021). All the other related species identified from BLAST searches produced ANI values below 95%.

In *Streptomyces*, MLSA genetic distance based on nucleotide sequences of five housekeeping genes is also considered a cutoff point for delineation of species within the genus. When the MLSA genetic distance between two sequences exceeds 0.007 the sequences are considered to belong to different species and vice versa (Rong & Huang, 2014). According to this, the close relationship between RMIT01 and *Streptomyces* YPW6 was further confirmed with an MLSA genetic distance of 0.00521 (Table S2). In contrast, other closely related species exhibited higher MLSA genetic distance values. Therefore, *Streptomyces* sp YPW6 isolated from a sediment sample collected from Wenzhou, China can be identified as the closest to the strain RMIT01.

In comparison with other closely related species, RMIT01 has medium length spore chain. *S. parvus* and *S. badius* reported spore chains with 10–50 spores, while *S. sindenensis* reported chains of 3–10 spores (Shirling & Gottlieb, 1968a; Shirling & Gottlieb, 1968b; Shirling & Gottlieb, 1972). A distinct feature of RMIT01 is its square shaped spores (Fig. 1), while other related species are reported to produce rod shaped spores (Table S1). *Streptomyces nodosus* has similarly shaped spores; however, its spore chain form tightly knotted spirals, which were absent in RMIT01. Similar to RMIT01, all related species were able to utilise D-mannitol as sole carbon source, while none showed growth on D-saccharose or inositol (Table S1). RMIT01 showed no growth on media containing D-glucose or D-fructose as the sole carbon source, whereas all other related species displayed growth on these substrates, indicating differences in carbon source utilisation (Shirling & Gottlieb, 1968a; Shirling & Gottlieb, 1968b; Shirling & Gottlieb, 1972). Consistent with RMIT01, *Streptomyces clavuligerus* NRRL 3585 also lacks the ability to utilise glucose as a sole carbon source (Garcia-Dominguez, Martin & Liras, 1989).

The aerial and substrate mycelium colours of RMIT01 described in the Table S1 and align closely with those of *S. griseus* (Shirling & Gottlieb, 1968a). *S. griseus* showed pale yellow or pale greenish yellow aerial spore mass colour and pale yellow to olive brown or light yellowish brown substrate mycelium colours (Table S1). Other closely related species mostly reported yellow colour range, with greenish yellow and orange colour being rare. Overall, the aerial and substrate mycelia colour of RMIT01 is more similar to *S. griseus*. None of the considered *Streptomyces* species reported substrate mycelial pigment colours.

RMIT01 exhibited antifungal activity against *C. albicans*. Optimization experiments showed that media composition, salt composition, incubation temperature and incubation time strongly influence its antifungal production. Furthermore, aerobic respiration also affected antifungal production, whereas the initial pH of the medium had no significant effect.

In incubation time optimization experiments, the largest antifungal inhibition zone was observed at the six day of incubation. This result was consistent with previous studies on different *Streptomyces* species, which indicate that bacterial biomass is generated during the first 1–5 days of incubation, while secondary metabolite production occurs between 5–12 days, depending on the species (Hu et al., 2024; Lertcanawanichakul & Sahabuddeen, 2023; Yang et al., 2025).

The optimum temperature of RMIT01 for antifungal production was 25 °C, which produced significantly larger inhibition zone compared to 20 °C and 30 °C. This result is consistent with Yang et al. (2025) who reported 25 °C as the optimal temperature for antifungal metabolite production. Although RMIT01 showed strong growth at 37 °C, no antifungal inhibition was detected, within the level of sensitivity of our detection method. On SC media, bacterial growth occurred between 20 °C and 40 °C; however, antifungal production was limited to an incubation range of 20–30 °C.

Regarding media type optimization, SC media produced a significantly larger antifungal inhibition zone compared to other media types (M1, ISP2, and Ismet), while the latter three did not differ significantly from one another. When comparing the other tested media with SC, the key difference was the nitrogen source: SC contained casein, whereas the other media used yeast extract as the common nitrogen source. Soluble starch was included as a carbon source in SC, M1 and Ismet, while glucose was present in ISP2 and Ismet. In the study by Lertcanawanichakul & Sahabuddeen (2023) using *Streptomyces* sp. KB1, the second highest inhibition zone against *Staphylococcus aureus* TISTR 517 for both experiments that study the carbon source and nitrogen source were observed with starch and casein added to LB/2 respectively. Also, Shakeel et al. (2016) reported similar results with a study conducted for optimization of the culture medium and conditions for production of antifungal substances by *Streptomyces platensis*. Previous studies have showed that rapidly metabolizing carbon sources such as glucose can reduce or inhibit secondary metabolite production (Escalante et al., 1982; Lertcanawanichakul & Sahabuddeen, 2023; Romero-Rodríguez et al., 2017). However, as non-mobile bacteria, *Streptomyces* species cannot actively seek a specific nutrient source and are adapted to utilise a variety of substrates (Krysenko & Wohlleben, 2024). Moreover, the preferred carbon and nitrogen sources may depend on the *Streptomyces* species, the specific biosynthetic pathway involved in the secondary metabolite, and the availability of building blocks supplying essential elements (Krysenko & Wohlleben, 2024).

Among the salt mixture tested, the Sigma™ sea salt mixture produced a significant increase in antifungal activity compared to the other two salt formulations. In some experiments, the presence of individual salts negatively affected secondary metabolite production when tested separately. For example, Lertcanawanichakul & Sahabuddeen (2023) reported that FeSO_4_, KCl, K_2_HPO_4_, and KNO_3_ influenced secondary metabolite production depending on the compound of interest. The study on *S. platensis* reported that K_2_HPO_4_, KH_2_PO_4_, and MgSO_4_ positively affected both cell growth and antifungal production, where MnCl_2_ and ZnCl_2_ had a negative effect on antifungal production (Shakeel et al., 2016). It is important to note that this experiment did not investigate the effect of each element and varying the concentration of each individual element on antifungal production. Although enhanced antifungal activity was observed when RMIT01 was grown in Sigma™ salt-supplemented medium compared with the other two salt formulations, the current literature does not provide a sufficient explanation for this increase in antifungal production. The observed enhancement may be related to the balanced ionic composition, appropriate osmotic strength and presence of biologically relevant trace elements in Sigma™ sea salt, which could promote secondary metabolism in this species. However, further studies targeting individual salt components and their concentrations are needed to confirm this hypothesis.

When differentiating initial pH levels of growth media (SC), antifungal activity was observed across all tested pH levels (pH 4.5–9). The negative control, containing no RMIT01 inoculum, did not exhibit inhibition zones, confirming that the medium’s pH alone did not affect *C. albicans*. Previous literature indicates that the optimal medium pH for secondary metabolite production in *Streptomyces* is typically around neutral values (Lertcanawanichakul & Sahabuddeen, 2023).

The results demonstrated that cultures grown under aerobic conditions exhibited stronger growth and higher antifungal production compared to those grown under anaerobic conditions. Notably, most previous experiments on *Streptomyces* metabolite production optimization have focused on aerobic conditions, often using agitation, while anaerobic conditions have been largely unexplored (Augustine et al., 2004; Lertcanawanichakul & Sahabuddeen, 2023; Shakeel et al., 2016). This experiment assessed antifungal production under anaerobic conditions, considering the water-submerged environments frequently experienced by mangrove rhizospheric microbes. Furthermore, previous research has shown that agitation at 100–200 rpm often enhances *Streptomyces* metabolite production (Augustine et al., 2004; Lertcanawanichakul & Sahabuddeen, 2023). As this study aimed to establish optimal conditions for cultures on solid media for subsequent experiments, agitation was not assessed.

Some of the BGCs identified in RMIT01 by AntiSMASH correspond to clusters previously reported to encode antifungal compounds, including thiazostatin, watasemycin A, watasemycin B, 2-hydroxyphenylthiazoline enantiopyochelin, isopyochelin (Sasaki et al., 2002), bafilomycin B1 (Pan et al., 2015; Zhang et al., 2013), naringenin (Huang et al., 2010), members of Heat-Stable Antifungal Factor family (Yu et al., 2007), hopene (Kongue et al., 2013), desulfoclethramycin, clethramycin (Cai et al., 2007), and 14-hydroxyisochainin (Li et al., 1989). Among these, the thiazostatin-watasemycin cluster is noteworthy, as the stereoisomers watasemycins A and B produced by *Streptomyces* sp. TP-A0597 have been shown to exert mild antifungal activity (100 µg/mL) against *C. albicans* A9540 and *C. tropicalis* IFO 1400 (Sasaki et al., 2002). In RMIT01, this cluster exhibited only medium similarity to the known BGC and it is not present among other related species. In addition, a BGC found in RMIT01 showed low similarity to the desulfoclethramycin/clethramycin gene cluster, previously reported to produce the compound clethramycin, which displays antifungal activity against yeasts and filamentous fungi (Cai et al., 2007). This BGC was found to be unique to RMIT01 amongst the interrogated species. This suggests that if the antifungal compounds detected in RMIT01 originate from this cluster, they may be structurally distinct from known compounds. The uniqueness of this BGC compared with those of closely related *Streptomyces* species highlights the potential of RMIT01 as a promising candidate for further studies, particularly compound isolation.

Compounds such as bafilomycin B (cluster bafilomycin B1), isosakuranetin and sakuranetin which were derivatives of naringenin (cluster naringenin), dihydromaltophilin (cluster Heat Stable Antifungal Factor (HSAF)), sonhafouonic acid (cluster hopene), and 14-hydroxyisochainin (cluster 14-hydroxyisochainin) have been reported to display antifungal activity against filamentous fungi (Huang et al., 2010; Kongue et al., 2013; Li et al., 1989; Pan et al., 2015; Yu et al., 2007; Zhang et al., 2013). Among these, dihydromaltophilin reported a novel mode of action by disrupting the biosynthesis of a distinct group of sphingolipids (Yu et al., 2007).

## Conclusions

This study was undertaken to characterize the *Streptomyces* sp. RMIT01, evaluate its antifungal potential, and explore its biosynthetic capacity through genome mining. RMIT01 is likely a novel species, as indicated by its unique morphological, biochemical and genomic characteristics. Further work to formally name the species is needed. It shows promise as an antifungal-producing strain. Optimal antifungal production was achieved in starch casein medium supplemented with Sigma™ sea salt, incubated at 25 °C for 6 days under aerobic conditions with antifungal testing against *C. albicans* revealing an average inhibition zone diameter of 20.9 mm. BGCs such as tyhiazostatin, bafilomycin B1, naringenin, HSAF, hopene, desulfoclethramycin/clethramycin, and 14-hydroxyisochainin were identified as potential contributors to its antifungal activity. Among them, the BGCs for thiazostatin, watasemycin A and B, 2-hydroxyphenylthiazoline, enantiopyochelin and isopyochelin, as well as desulfoclethramycin and clethramycin, showed medium to low similarity to known BGCs and may have the potential to produce structurally novel compounds. Further investigations such as bioassay guided fractionation is recommended to identify the specific compound(s) responsible for the antifungal effect.

##  Supplemental Information

10.7717/peerj.20901/supp-1 Supplemental Information 1 Phenotypic and biochemical characteristics differentiating strain RMIT01 from other related species of the genus *Streptomyces* + Positive results - Negative results ND, No Data; S, Susceptible

10.7717/peerj.20901/supp-2 Supplemental Information 2 MLSA evolutionary distances between *Streptomyces* sp. RMIT01 and closely related species * Indicates the lowest value

10.7717/peerj.20901/supp-3 Supplemental Information 3 Statistical summary of incubation conditions vs. antifungal inhibition zone diameter in *Streptomyces* sp. RMIT01 NS, Not significant; NA, Not applicable

## Additional Information and Declarations

### Data Availability

The following information was supplied regarding data availability: The genome sequence of RMIT01 strain is available at NCBI GenBank: CP194014. The raw data is available in the Supplemental Files.
