# Supplementary material for: Characterization and optimization of antifungal production in Streptomyces sp. RMIT01 from the Australian mangrove rhizosphere
Source: PeerJ. 2026 May 4;14:e20901. doi: 10.7717/peerj.20901 (PMC13151935; doi:10.7717/peerj.20901)
Supplement: Supplemental Information 1 — + Positive results - Negative results ND, No Data; S, Susceptible [file peerj-14-20901-s001.docx]

**Table S1:** Phenotypic and biochemical characteristics differentiating strain RMIT01 from other related species of the genus *Streptomyces*

| **Strain** | | | **RMIT01** | ***S. badius* ISP 5139 (Shirling & Gottlieb 1968b)** | ***S. parvus* NRRL-B-1455 (Shirling & Gottlieb 1972)** | ***S. sindenensis***  **ISP 5255 (Shirling & Gottlieb 1968a)** | ***S. bacillaris* INMI 445 (Shirling & Gottlieb 1972)** | ***S. griseus* IMRU 3463 (Shirling & Gottlieb 1968a)** |
| --- | --- | --- | --- | --- | --- | --- | --- | --- |
| **Spores per chain (n)** | | | 15-20 | 10-50 | 10-50 | 3-10 | 10-50 | 10-50 |
| **Spore shape** | | | Square shape, formed by fragmentation of arial mycelium, smooth spores on oatmeal agar 14 days | Rod shape, formed by fragmentation of arial mycelium, smooth spores on oatmeal agar 14 days | Rod shape, smooth spores on oatmeal agar 26-days | Rod shape, smooth spores on yeast-malt agar, oatmeal agar, salts starch agar and glycerol-asparagine agar 14 days | Rod shape, smooth spores on yeast-malt agar, oatmeal agar, salts starch agar and glycerol-asparagine agar 14 days | Rod shape, smooth spores on yeast-malt agar, oatmeal agar, salts starch agar and glycerol-asparagine agar 14 days |
| **ISP2** (Yeast extract-malt agar) | | **Arial spore mass colour** | Light yellow to pale yellow | Yellow colour-series | Pale yellow | Grayish  yellowish pink | Pale yellow Some parts were white or light grayish yellowish  brown | Pale yellow or pale greenish yellow |
|  |  | **Substrate mycelium pigments** | No pigments, yellowish white | No pigments | No pigments, light yellow to light greenish yellow | No pigments, Pale grayish yellow. | No pigments  orange-yellow to yellowish brown | No pigments, grayed yellow to  olive brown or light yellowish brown |
| **ISP3** (Oatmeal agar) | | **Arial spore mass colour** | Moderate olive to pale greenish yellow | Yellow colour-series | Pale yellow | White or yellow colour-series | Pale yellow | Pale yellow or pale greenish yellow |
|  |  | **Substrate mycelium pigments** | No pigments, greyish greenish yellow - yellowish white | No pigments | No pigments, light yellow to light greenish yellow | No pigments, Pale grayish yellow. | No pigments, light grayish yellow | No pigments, grayed yellow to  olive brown or light yellowish brown |
| **ISP4** (Inorganic salts-starch agar) | | **Arial spore mass colour** | Moderate greenish yellow- yellowish white | Yellow colour-series | Pale yellow | White colour series | Pale yellow | Pale yellow or pale greenish yellow |
|  |  | **Substrate mycelium pigments** | No pigments | No pigments | No pigments, light yellow to light greenish yellow | No pigments, Pale grayish yellow. | No pigments, yellowish or orange yellow | No pigments, grayed yellow to  olive brown or light yellowish brown |
| **ISP5** (Glycerol-asparagine agar) | | **Arial spore mass colour** | Greyish green yellow to yellowish white | Yellow colour-series | Pale yellow | White colour series | Pale yellow | Pale yellow or pale greenish yellow |
|  |  | **Substrate mycelium pigments** | No pigments, greyish green yellow | No pigments | No pigments, light yellow to light greenish yellow | Pale grayish yellow. No pigments | No pigments, yellowish or orange yellow | No pigments, grayed yellow to  olive brown or light yellowish brown |
| Temperature range for growth (°C) 4, 10, 15, 20, 25, 28, 35, 37, 40, and 45 ◦C | | | 15- 37 ^o^C | ND | ND | ND | ND | ND |
| pH range (pH 3.0–12.0, at intervals of 1.0 pH unit) | | | 6-11 | ND | ND | ND | ND | ND |
| Tolerance of NaCl (%, w/v) NaCl tolerance (0%–15% (w/v) in 1% intervals) | | | 0 – 10% | ND | ND | ND | ND | ND |
| **Assimilation of sole carbon sources** | | | | | | | | |
| 1 | GLY | Glycerol | - | ND | ND | ND | ND | ND |
| 2 | ERY | Erythritol | - | ND | ND | ND | ND | ND |
| 3 | DARA | D-arabinose. | - | ND | ND | ND | ND | ND |
| 4 | LARA | L-arabinose | - | + | + | + | - | - |
| 5 | RIB | D-ribose | - | ND | ND | ND | ND | ND |
| 6 | DXYL | D-xylose | + | + | + | + | - | + |
| 7 | LXYL | L-xylose | - | ND | ND | ND | ND | ND |
| 8 | ADO | D-Adonitol | - | ND | ND | ND | ND | ND |
| 9 | MDX | Methyl-beta-D-xylopyranoside | - | ND | ND | ND | ND | ND |
| 10 | GAL | D-galactose | - | ND | ND | ND | ND | ND |
| 11 | GLU | D-glucose | - | + | + | + | + | + |
| 12 | FRU | D-fructose | - | + | + | + | + | + |
| 13 | MNE | D-mannose | + | ND | ND | ND | ND | ND |
| 14 | SBE | L-sorbose | - | ND | ND | ND | ND | ND |
| 15 | RHA | L-rhamnose | - | - | + | - | - | - |
| 16 | DUL | Dulcitol | - | ND | ND | ND | ND | ND |
| 17 | INO | Inositol | - | - | - | - | - | - |
| 18 | MAN | D-mannitol | + | + | + | + | + | + |
| 19 | SOR | D-sorbitol | - | ND | ND | ND | ND | ND |
| 20 | MDM | Methyl-alpha-D-mannopyranoside | - | ND | ND | ND | ND | ND |
| 21 | MDG | Methyl-alpha-D-glucopyranoside | - | ND | ND | ND | ND | ND |
| 22 | NAG | N-acetylglucosamine | - | ND | ND | ND | ND | ND |
| 23 | AMY | Amygdalin | - | ND | ND | ND | ND | ND |
| 24 | ARB | Arbutin |  | ND | ND | ND | ND | ND |
| 25 | ESC | Esculin ferric citrate | + | ND | ND | ND | ND | ND |
| 26 | SAL | Salicin | - | ND | ND | ND | ND | ND |
| 27 | CEL | D-cellobiose | - | ND | ND | ND | ND | ND |
| 28 | MAL | D-maltose | - | ND | ND | ND | ND | ND |
| 29 | LAC | D-lactose (bovine origin) | - | ND | ND | ND | ND | ND |
| 30 | MEL | D-melibiose | - | ND | ND | ND | ND | ND |
| 31 | SAC | D-saccharose (sucrose) | - | - | **-** | - | - | - |
| 32 | TRE | D-trehalose | - | ND | ND | ND | ND | ND |
| 33 | INU | Inulin | - | ND | ND | ND | ND | ND |
| 34 | MLZ | D-melezitose | - | ND | ND | ND | ND | ND |
| 35 | RAF | D-raffinose | - | - | - | - | - | - |
| 36 | AMD | Amidon (starch) | - | ND | ND | ND | ND | ND |
| 37 | GLYG | Glycogen | - | ND | ND | ND | ND | ND |
| 38 | XLT | Xylitol | - | ND | ND | ND | ND | ND |
| 39 | GEN | Gentiobiose | - | ND | ND | ND | ND | ND |
| 40 | TUR | D-turanose | - | ND | ND | ND | ND | ND |
| 41 | LYX | D-lyxose | - | ND | ND | ND | ND | ND |
| 42 | TAG | D-tagatose | - | ND | ND | ND | ND | ND |
| 43 | DFUC | D-fucose | - | ND | ND | ND | ND | ND |
| 44 | LFUC | L-fucose | - | ND | ND | ND | ND | ND |
| 45 | DARL | D-arabitol | + | ND | ND | ND | ND | ND |
| 46 | LARL | L-arabitol | - | ND | ND | ND | ND | ND |
| **Nitrogen source (utilization of sole nitrogen sources)** | | | | | | | | |
| L-cysteine | | | + | - | - | ND | ND | ND |
| L-lysine | | | + | - | - | ND | ND | ND |
| L-glycine | | | + | - | - | ND | ND | ND |
| L-glutamine | | | + | - | - | ND | ND | ND |
| L-valine | | | + | - | - | ND | ND | ND |
| L-arginine | | | + | - | - | ND | ND | ND |
| L-asparagine | | | + | - | - | ND | ND | ND |
| **Biochemical characterisation** | | | | | | | | |
| Liquefaction of gelatine | | | - | - | - | ND | ND | ND |
| Hydrolysis of starch | | | - | - | - | ND | ND | ND |
| Reduction of nitrate | | | - | ND | ND | ND | ND | ND |
| Production of H_2_S | | | - | ND | ND | ND | ND | ND |
| 3% H_2_O_2_ | | | + | ND | ND | ND | ND | ND |
| Hydrolyzation of Tween 20 | | | + | ND | ND | ND | ND | ND |
| Hydrolyzation of Tween 80 | | | + | ND | ND | ND | ND | ND |
| Tetracycline | | | S | ND | ND | ND | ND | ND |
| Kanamycin | | | S | ND | ND | ND | ND | ND |
| Streptomycin | | | S | ND | ND | ND | ND | ND |
| Lincomycin | | | S | ND | ND | ND | ND | ND |
| Vancomycin | | | S | ND | ND | ND | ND | ND |

+ Positive results

- Negative results

ND: No Data

S: Susceptible
