# Supplementary material for: Characterization and optimization of antifungal production in Streptomyces sp. RMIT01 from the Australian mangrove rhizosphere
Source: PeerJ. 2026 May 4;14:e20901. doi: 10.7717/peerj.20901 (PMC13151935; doi:10.7717/peerj.20901)
Supplement: Supplemental Information 2 — * Indicates the lowest value [file peerj-14-20901-s002.docx]

**Table S2:** MLSA evolutionary distances between *Streptomyces* sp. RMIT01 and closely related species

| **Species** | **Whole genome/ housekeeping genes (NCBI Reference Sequence/ GenBank** | **MLSA evolutionary distance for strain RMIT01** |
| --- | --- | --- |
| *Streptomyces* sp. YPW6 | NZ_CP076457.1 | 0.00521***** |
| *S. bacillaris* ATCC 15855 | CP029378.1 | 0.04231 |
| *S. griseus* JCM 4516 | BNBJ00000000.1 | 0.03515 |
| *S. parvus* JCM 4069 | NZ_BMRY00000000.1 | 0.02598 |
| *S. mangrovisoli* MUSC 149 | LAVA00000000.2 | 0.01185 |
| *S. cyaneofuscatus* NRRL B-2570 | JOEM00000000.1 | 0.07380 |
| *S. rubiginosohelvolus* JCM 4415 | BMTW00000000.1 | 0.03138 |
| *S. fulvorobeus* DSM 41455 | JACCCF000000000.1 | 0.07219 |
| *S. arboris* TRM68085 | VYUA00000000.1 | 0.05907 |
| *S. cavourensis* DSM 41795 | VFRC00000000.1 | 0.04402 |
| *S. parvus* NRRL B-1455 | VXCD00000000.1 | 0.02598 |
| *S. mediolani* NRRLWC 3934 | JOJK00000000.1 | 0.03181 |
| *S. violaceoruber* NRRL B-2935^T^ | atpD - KT384751.1, gyr B - KT385099.1, rec A - KT385453.1, rpo B - KT389071.1, trp B - KT389420.1 | 0.10539 |
| *S. sindenensis* AS 4.626^T^ | atpD - EF031274.1, gyr B - EF054963.1, rec A - EF055016.1, rpo B - EF055071.1, trp B - EF055126.1 | 0.02641 |
| *S. sindenensis* JCM 4164^T^ | BMSG00000000.1 | 0.02641 |
| *S. californicus* NRRL B-2098^T^ | JNZZ00000000.1 | 0.03979 |
| *S. rhizosphaericola* 1AS2c^T^ | SRZK00000000.1 | 0.04443 |
| *S. globisporus* NRRLB 2872^T^ | atpD - KT384568.1, gyr B - KT384917.1, rec A - KT385266.1, rpo B - KT388887.1, trp B - KT389237.1 | 0.03223 |
| *S. flaveolus* JCM 4032^T^ | BMRV00000000.1 | 0.11661 |
| *S. badius* JCM 4350 ^T^ | BMSZ00000000.1 | 0.02766 |
| *S. durocortorensis* RHZ10^T^ | JAFEUF010000614.1 | 2.44475 |

*****Indicates the lowest value
