# Supplementary material for: Characterization and optimization of antifungal production in Streptomyces sp. RMIT01 from the Australian mangrove rhizosphere
Source: PeerJ. 2026 May 4;14:e20901. doi: 10.7717/peerj.20901 (PMC13151935; doi:10.7717/peerj.20901)
Supplement: Supplemental Information 3 — NS, Not significant; NA, Not applicable [file peerj-14-20901-s003.docx]

**Table S3:** Statistical summary of RMIT01 incubation conditions vs. antifungal inhibition zone diameter with Mean, SD, and 95% CI

| **Parameter** | **Treatment** | **Test** | **Test Statistic** | **Degrees of Freedom** | ***p*-value** | **Inhibition diameter (mm)** | **Mean (mm)** | **SD** | **95% CI Lower** | **95% CI Upper** | **Turkey’s Multiple Mean Comparison** |
| --- | --- | --- | --- | --- | --- | --- | --- | --- | --- | --- | --- |
| Incubation Time | Day 3 | One-way ANOVA | F (7, 16) = 3.570 | 7, 16 | 0.0166 | R1 – 13.5  R2 – 14.8  R3 – 14.5 | 14.3 | 0.681 | 12.576 | 15.958 | NS |
|  | Day 4 |  |  |  |  | R1 – 14.0  R2 – 14.0  R3 – 11.5 | 13.2 | 1.443 | 9.581 | 16.752 | vs Day 6 (*p*=0.0130) |
|  | Day 5 |  |  |  |  | R1 – 14.0  R2 – 14.5  R3 – 14.5 | 14.3 | 0.289 | 13.616 | 15.05 | NS |
|  | Day 6 |  |  |  |  | R1 – 14.5  R2 – 17.0  R3 – 17.0 | 16.2 | 1.443 | 12.581 | 19.752 | vs Day 4 (*p*=0.0130)  vs Day 7 (*p*=0.0255)  vs Day 8 (*p*=0.0255) |
|  | Day 7 |  |  |  |  | R1 – 13.0  R2 – 13.0  R3 – 14.2 | 13.4 | 0.693 | 11.679 | 15.121 | vs Day 6 (*p*=0.0255) |
|  | Day 8 |  |  |  |  | R1 – 12.8  R2 – 13.8  R3 – 13.8 | 13.5 | 0.577 | 12.032 | 14.901 | vs Day 6 (*p*=0.0255) |
|  | Day 9 |  |  |  |  | R1 – 13.2  R2 – 12.5  R3 – 14.0 | 13.23 | 0.751 | 11.369 | 15.098 | NS |
|  | Day 10 |  |  |  |  | R1 – 13.8  R2 – 15.0  R3 – 14.0 | 14.27 | 0.640 | 12.670 | 15.860 | NS |
| Incubation Temperature | 40^o^C |  | F (5,12) = 3671 | 5,12 | <0.0001 | R1 – 0.0  R2 – 0.0  R3 – 0.0 | 0.00 | 0.000 | 0.000 | 0.000 | vs 20^o^C (*p*<0.0001)  vs 25^o^C (*p*<0.0001)  vs 30^o^C (*p*<0.0001) |
|  | 37^o^C |  |  |  |  | R1 – 0.0  R2 – 0.0  R3 – 0.0 | 0.00 | 0.000 | 0.000 | 0.000 | vs 20^o^C (*p*<0.0001)  vs 25^o^C (*p*<0.0001)  vs 30^o^C (*p*<0.0001) |
|  | 30^o^C |  |  |  |  | R1 – 16.0  R2 – 16.8  R3 – 16.2 | 16.3 | 0.416 | 15.299 | 17.368 | vs 20^o^C (*p*=0.0196)  vs 25^o^C (*p*=0.0001) |
|  | 25^o^C |  |  |  |  | R1 – 17.8  R2 – 18.0  R3 – 18.0 | 17.9 | 0.115 | 17.646 | 18.22 | vs 20^o^C (*p*<0.0001)  vs 30^o^C (*P*<0.0001) |
|  | 20^o^C |  |  |  |  | R1 – 16.0  R2 – 15.0  R3 – 15.5 | 15.5 | 0.500 | 14.258 | 16.742 | vs 25^o^C (*p*=0.0001)  vs 30^o^C (*p*=0.0196) |
|  | 15^o^C |  |  |  |  | R1 – 0.0  R2 – 0.0  R3 – 0.0 | 0.00 | 0.000 | 0.00 | 0.00 | vs 20^o^C (*p*<0.0001)  vs 25^o^C (*p*<0.0001)  vs 30^o^C (*p*<0.0001) |
| Media Type | Starch Casein |  | F (3, 8) = 45.28 | 3, 8 | <0.0001 | R1 – 21.2  R2 – 20.5  R3 – 21.0 | 20.9 | 0.361 | 20.004 | 21.796 | vs ISP2 (*p*=<0.0001)  vs Ismet (*p*=0.0003)  vs M1 (*p*=<0.0001) |
|  | ISP2 |  |  |  |  | R1 – 15.0  R2 – 16.5  R3 – 16.0 | 15.8 | 0.764 | 13.936 | 17.731 | NS |
|  | Ismet |  |  |  |  | R1 – 16.2  R2 – 17.5  R3 – 17.5 | 17.1 | 0.751 | 15.202 | 18.931 | NS |
|  | M1 |  |  |  |  | R1 – 16.0  R2 – 16.0  R3 – 15.0 | 15.7 | 0.577 | 14.232 | 17.101 | NS |
| Salt Composition | Sigma^TM^ sea salts |  | F (2, 6) = 22.79 | 2, 6 | 0.0016 | R1 – 18.2  R2 – 18.8  R3 – 18.5 | 18.5 | 0.300 | 17.755 | 19.245 | vs Artificial Sea Salt (*p*=0.0046)  vs Tropic Marine Salts (*p*=0.0018) |
|  | Tropic Marine^TM^ |  |  |  |  | R1 – 17.2  R2 – 17.1  R3 – 17.4 | 17.2 | 0.153 | 16.854 | 17.613 | NS |
|  | Artificial sea salt |  |  |  |  | R1 – 17.5  R2 – 17.8  R3 – 17.1 | 17.5 | 0.351 | 16.594 | 18.339 | NS |
| pH | pH4.5 |  | F (5, 12) = 0.7764 | 5, 12 | 0.5851 | R1 – 17.8  R2 – 17.5  R3 – 17.8 | 17.7 | 0.173 | 17.270 | 18.130 | NS |
|  | pH5 |  |  |  |  | R1 – 16.2  R2 – 17.8  R3 – 18.5 | 17.5 | 1.179 | 14.571 | 20.429 | NS |
|  | pH6 |  |  |  |  | R1 – 17.8  R2 – 18.2  R3 – 18.2 | 18.1 | 0.231 | 17.493 | 18.640 | NS |
|  | pH7 |  |  |  |  | R1 – 17.2  R2 – 18.0  R3 – 18.0 | 17.7 | 0.462 | 16.586 | 18.881 | NS |
|  | pH8 |  |  |  |  | R1 – 17.2  R2 – 17.5  R3 – 17.5 | 17.4 | 0.173 | 16.970 | 17.830 | NS |
|  | pH9 |  |  |  |  | R1 – 17.4  R2 – 17.0  R3 – 17.6 | 17.3 | 0.306 | 16.574 | 18.092 | NS |
| Aerobic vs Anaerobic | Aerobic | Unpaired t-test | t = 10.45 | 4 | 0.0002 | R1 – 18.2  R2 – 18.5  R3 – 19.2 | 18.6 | 0.513 | 17.359 | 19.908 | NA |
|  | Anaerobic |  | t = 10.45 | 4 | 0.0002 | R1 – 12.8  R2 – 13.5  R3 – 14.1 | 13.5 | 0.651 | 11.850 | 15.083 | NA |

NS – Not significant

NA – Not applicable
